# Effective forewarning requires central route processing: Theoretical improvements on the counterargumentation hypothesis and practical implications for scam prevention

**DOI:** 10.1371/journal.pone.0229833

**Published:** 2020-03-05

**Authors:** Yasuhiro Daiku, Naoki Kugihara, Tsukasa Teraguchi, Eiichiro Watamura

**Affiliations:** 1 Graduate School of Human Sciences, Osaka University, Osaka, Japan; 2 Japan Society for the Promotion of Science, Tokyo, Japan; 3 Department of Food and Nutrition, Higashi Chikushi Junior College, Fukuoka, Japan; Fordham University, UNITED STATES

## Abstract

Financial scams have caused tremendous financial damage globally. In Japan, the police forewarn people by equipping them with scam-prevention techniques or providing awareness regarding examples of previous scams; however, this does not appear to effectively prevent the damage, as many scam victims do not remember these warnings when faced with actual scam encounters. Considering that scammers often use appeal to emotion techniques, peripheral processing during scam attempts might disturb people’s abilities to recall the warnings on scammers’ modus operandi, thus leading to failed counter-arguing efforts. We verified this hypothesis in an experimental setting by asking 162 participants to remember given forewarnings and resist deceptive advertisements. The results showed that participants gave the advertisers’ manipulative intent a higher rating only when they processed the advertisement through a central route, in addition to being forewarned. This means that forewarning had no effect when participants processed the advertisement through a peripheral route. Moreover, forewarning recollection levels mediated the effect of processing route on this rating, which suggests that remembering forewarnings is necessary to generate counterarguments. This result expands the theory on forewarning effects and explains why people are susceptible to scam victimization. Furthermore, it provides implications for scam prevention.

## Introduction

Financial scams have been a critical social problem in Japan for several years. In 2016, the amount of financial damage caused by scams in Japan amounted to around 40 billion yen [1]. The police, banks, and the government have tried to solve this issue using various strategies, such as limiting maximum bank transfer amounts and issuing scam warnings [2]. However, scams continue to be a major concern, and threaten individuals’ property and well-being. To make matters worse, scams are prevalent not only in Japan, but also worldwide [3].

The most common way to prevent scams is by preemptively warning potential victims. This is called “forewarning,” in social psychology contexts, and is defined as offering information on impending appeals, such as topics, stances, or intents to persuade [4, 5]. Persuasion research has studied forewarning mechanisms extensively and has found forewarnings sufficient to elicit resistance [6].

In recent years, some researchers have begun to apply the substantial findings to real-world persuasion resistance [7–9]. For example, Scheibe et al. [9] investigated the effectiveness of forewarning in a scam situation. First, they selected participants without obtaining their consent by cooperating with the US Postal Inspection Service and a commercial company. Then, well-trained volunteers from the American Association of Retired Persons (AARP) Fraud Fighter Call Center forewarned the participants on the phone and, a few weeks later, they received a mock fraud call from a research assistant with prior experience as a professional telemarketer. The researchers recorded the participants’ responses and found that the forewarning strengthened their resistance against the mock scam. This result aligned with AARP’s prior similar work [7] that revealed analogous results.

These experimental and applied forewarning studies suggest the effectiveness of forewarning in preventing individuals from being defrauded by scams. However, the fact remains that most real scam victims in Japan were forewarned before they were defrauded. According to a survey by the National Police Agency [10], 70.7% of scam victims were aware of the scammers’ modus operandi. For the majority of victims, forewarning did not prevent them from being scammed, which is greatly inconsistent with previous studies’ results [7–9].

What causes this discrepancy? One important suggestion originated with Petty and Cacioppo [11], who investigated the effect of personal involvement on forewarning efficiency. They found that forewarning was more effective when participants felt highly involved in the topic. In short, personal involvement adjusted the forewarning’s effect, as also revealed by Wood and Quinn’s review [6]. In a subsequent study, Petty and Cacioppo expanded on and factored this result into their elaboration likelihood model (ELM) [12]. ELM assumes that both motivation and processing abilities are essential for managing central routes. In the model, involvement is a factor that determines motivation levels. From the ELM perspective, peripheral route processing may weaken the forewarning effect in real scam-attempt situations, as scammers often use appeal to emotion techniques that can elicit this peripheral processing [13].

In persuasion research, Petty and Cacioppo’s counterargumentation hypothesis is the most influential theory on how this peripheral process weakens forewarning effects [14]. This hypothesis assumes that it is not the forewarning per se that produces persuasion resistance, rather it is the cognitive counterargumentation elicited during the temporal delay between the forewarning and the triggered resistance to the persuasion message. Therefore, peripheral route processing during the delay has been assumed to weaken the forewarning effect by disturbing counterargumentation. In fact, using distractions during the forewarning-message interval invalidates the forewarning effect [15]. In short, ELM assumes that disturbing the preparation process, between forewarning and persuasion, can weaken the forewarning’s effects.

However, this model does not appropriately apply to real-world scam resistance, because it depends on a process that does not function in actuality. In most previous experimental research, including those on counterargumentation theory, participants were told that they would be exposed to a persuasion message when being forewarned. In reality, one cannot predict whether forewarned people will encounter scam attempts or not. Therefore, counterargumentation theory’s assumed preparation process, in which people generate counterarguments to appeals, never occurs in reality. Instead, when scamming attempts take place, people must “remember” the forewarning and then generate counterarguments during the message presentation to resist without prepared counterarguments.

This real-world resistance requires two new kinds of processing: remembering forewarnings and counterargumentation during message presentation. Only Petty and Cacioppo [11] tackled this issue and indicated that peripheral route processing weakens forewarning effects by disturbing counterargumentation not only during preparation time, but also during persuasion. They suggested that eliciting counterarguments can occur during as well as before message presentation.

However, no research has taken forgetting forewarnings into consideration, which may be the underlying factor in real-world resistance. In fact, 67.4% of scam victims reported not considering the possibility of a scam when being deceived [10]. This fact clearly indicates that most victims entirely forgot the forewarning about scammers’ modus operandi when experiencing the scam. Therefore, clarifying what disrupts forewarning remembrance during scamming is important for prevention.

In this study, we hypothesized that peripheral route processing disrupts forewarning remembrance as well as counterarguing during message presentation, based on memory studies' indications that acute stress disturbs memory retrieval [16]. In other words, the forewarning will not lead to counterarguing if it is forgotten. Furthermore, most previous research has adopted unrealistic experimental designs in which participants do not need to remember these forewarnings. Therefore, we invented a new experimental method, the “black background effect” scheme, that prevents participants from generating counterarguments before message presentation and provides the opportunity to remember the forewarning.

In summary, con artists’ emotionally manipulative methods are understood to spark peripheral processing and may prevent people from recalling forewarnings, resulting in a failure to resist scams. We hypothesized that peripheral processing would disturb forewarning remembrance and the resulting counterarguing process. In other words, for effective forewarning, central route processing is necessary.

## Method

All studies (preliminary survey, pilot experiment, and main experiment) were approved by the Osaka University’s School of Human Sciences’ ethics committee (27–059, 28–031). For all studies, all participants provided written consent. For the online study, we used a website to obtain consent.

### Preliminary survey and pilot study

Before the experiment, we conducted a preliminary survey and a pilot study to determine the appropriate types of stimuli and/or manipulations. First, the preliminary survey served to help us determine an appropriate persuasion message. Past research has illustrated that participants’ evaluations of messages change based on content and processing route. For example, when strong arguments are presented, centrally processing audiences have more message-congruent attitudes than peripherally processing audiences [17]. Considering these results, we chose a standard stimulus that introduces moderate arguments and whose evaluation was stable across the two processing routes. If message evaluations differed based solely on the processing route, we would be unable to test our hypothesis that assumed an interaction effect between forewarning and processing route, due to the emergence of another interaction effect between message content and processing route. Thus, we intended to create a stimulus message that included moderate arguments across the processing route, and averted ceiling and floor effects.

Second, after selecting an advertisement from the preliminary survey, we conducted a pilot study to confirm that our experimental manipulation was appropriate. University students participated in the pilot study, set in a laboratory, where we identified three problems in our procedure and manipulation of processing routes. Therefore, we revised these factors when conducting the final study. Preliminary survey and pilot study details are available in the online supplement (S1 File).

### Participants

We recruited 200 participants online through a crowdsourcing service site, who were offered 300 JPY each for completing the experiment. However, we were unable to record 15 participants’ data due to technical problems. Thus, we collected data from 185 participants (77 males and 108 females, *M*_*age*_ = 37.19±11.62, Range: 18–69).

### Design and procedure

We adopted a 2 (forewarning: forewarned or control) × 2 (processing route: central or peripheral) between-participants design (ANOVA). All experimental procedures, including condition manipulation, advertisement presentation, and dependent variable measurements, were performed on a PC. Participants were asked to download an Excel file that included a Visual Basic Application (VBA) code. The experiment, for which we obtained online written consent from the participants, began once they opened the file.

Participants were asked to answer a questionnaire that they were told was unrelated to the experiment. Instead, they were informed that the questionnaire was intended to make them practice using the program to answer questions. This questionnaire outlined six general psychological phenomena: misattribution of arousal, in group favoritism, the black background effect (or optimistic bias), the bystander effect, correspondence bias, and the Zeigarnik effect. The participants then rated the phenomena with one dichotomous item, “I know this phenomenon,” and two seven-point items, “I am interested in this phenomenon” and “I would like to learn more about this.” The forewarning condition was manipulated at that time through the fake psychology phenomenon, the black background effect, included in the six. Its explanation was as follows: “People generally become gullible when they encounter white letters used on a black background, because this combination makes people less perceptive. Therefore, people should be wary of this as some con artists use this technique.” However, participants in the control condition received an explanation of optimistic bias. This black background effect was related to the persuasion message they were going to read later, but were unaware of at that time.

Following the questionnaire, the participants received a fake explanation about the experiment, that it investigated the effects of task performance on buying behavior. To begin the experiment, they were asked to enter their sex, age, and whether they were native Japanese speakers. Thereafter, they read an on-screen advertisement, identical to our ad A from the preliminary survey (S1 File). The fake advertisement was for bottled water and contained white letters on a black background (the black background effect). It was presented for 60 seconds, at which time the processing route was manipulated by changing participants’ motivation and ability using the ELM. In the central processing condition, participants were asked to read the advertisement carefully, whereas in the peripheral processing condition, participants did not receive any specific instructions affecting their motivation and were asked to read the advertisement while answering a comparison calculation task (e.g. “Which is larger, 56–17 or 8×5?”). After reading the ad, the participants answered a related questionnaire. At the end of the experiment, the program debriefed the participants, we revealed our true aim, apologized for misleading them, and provided confirmation codes to submit online. We asked the participants to submit these codes, that appeared at the end of the program, and their Excel response files, which were then coded.

### Measurements

The participants rated the items for advertisement evaluations, processing route check and forewarning remembrance, and personalities, in this order.

#### Advertisement evaluations

We measured the average purchase intention using a seven-point Likert scale (1 = strongly disagree to 7 = strongly agree) for the following statements: “I would like to try the product,” “I will buy the product,” and “I will not buy the product (a reversal of the positive statement).” Similarly, we measured ad attitude items that included “The advertisement is good,” “I am comfortable with this advertisement,” and “I dislike the advertisement (reversal),” and product evaluation items that comprised “I prefer the product,” “I have a good impression of the product,” “I perceive the product as being of high quality,” and “The product is unattractive (reversal).” Lastly, the ad interest items included “I am interested in the advertisement,” “I am not interested in the advertisement (reversal),” and “I am attracted to the advertisement.” In addition, Campbell’s [18] inference of manipulative intent (IMI) was measured through an average of six seven-point items, for example, “The advertiser tried to manipulate the audience in ways that I did not like.” As we were interested in how people perceived a persuader’s deceptive intent, we mainly focused on IMI in the following analysis.

#### Processing route check and predicting questionnaire remembrance

To determine whether the manipulation changed the information processing route, we asked participants to indicate their level of elaboration as an average of three seven-point items: “I concentrated when reading the advertisement,” “I thought deeply about the content of the advertisement,” and “I did not read the advertisement carefully. (reversal).” Also in this section, we asked the participants to reflect on how much they remembered the previous questionnaire, before beginning the experiment, with two seven-point items: “I thought about the questionnaire while reading the advertisement” and “I linked the questionnaire to the advertisement.”

We subsequently conducted recognition and recall tests. In the recognition test, participants were asked to judge whether twelve psychological terms had been in the questionnaire they completed before the experiment. The twelve terms included six from the original questionnaire and six dummy terms. In the recall test, participants were given a free-writing task on the black background effect (forewarned condition) or optimism bias (control condition). Then, using a dichotomous scale, we asked them whether they were aware of the bottled water before the experiment started.

#### Personalities

The participants answered the Japanese version of the need for cognition scale [19] and the skepticism toward advertising scale (SKEP) [20]. Since we did not have an officially translated Japanese version of SKEP, we used the Japanese version translated by Igarashi [21]. In SKEP’s original version, higher scores mean that the evaluators are less skeptical of advertisements. To avoid confusion, we reversed the scores in the following analysis. We also measured participants’ personal involvement with bottled water as the average of four seven-point scales: “I usually buy bottled water,” “I like bottled water,” “I stick to a particular brand when I buy bottled water,” and “I usually do not buy bottled water, because I drink tap water (reversal).” We also added a measurement of their perceived vulnerability to ads, to measure their susceptibility to deception, as the average of four seven-point scales: “In daily life, I often feel I am being deceived by an advertisement,” “I do not think I am tempted by an advertisement (reversal),” “I think I can tell when people are forcing me to buy something (reversal),” “I can tell when there is exaggeration in an advertisement (reversal),” and “I am easily deceived by a tempting deal.” These items were measured to control the personal differences that may encourage central processing by giving motivation to process.

### Working hypothesis

As stated above, we hypothesized that the peripheral route prevents remembering forewarnings and leads to failed counterarguing, which also implies that the central route is necessary for effective forewarning against scam attempts. Therefore, participants processing through the central route would be more resistant to a persuasion message, whereas the opposite is true for those processing through the peripheral route. This discussion lead to more concrete hypotheses: Compared to the peripheral route, participants in the central route condition will infer the advertisement to be more manipulative than those in the peripheral route condition, but only when they are forewarned. This is mediated by the differences in the level of forewarning remembrance.

## Results

We excluded participants who were dishonestly engaging in the experiment, including those who ignored the comparison calculation task with one or zero answers (nine participants) and those who answered that they were aware of the black background effect (nine participants) or the imaginary bottled water in the advertisement (five participants, including one participant that claimed to know the black background effect as well). We also excluded one participant who reported searching online for the product in the advertisement during the experiment. Thus, we analyzed 162 participants’ data (64 male and 98 female participants, *M*_*age*_ = 36.44±11.28, Range: 18–65).

### Manipulation check of processing route

To test whether the experiment’s manipulation changed the information processing route, we ran a 2 × 2 ANOVA on the participant’s reported level of elaboration (*α* = .89). Only the main effect of the processing route emerged as significant, *F* (1, 158) = 96.62, *p* < .001, *partial η*^2^ = .38). Central-route processing respondents reported a higher level of elaboration than peripheral-route processing respondents (Table 1).

**Table 1:** Means (SDs) and ANOVA results of manipulation checks and ad evaluations.

	Forewarned		Not Forewarned		Significant Effects	
	Central	Peripheral	Central	Peripheral	ANOVA	Simple Effect Tests
Level of Elaboration	4.83 (1.41)	2.99 (1.46)	5.01 (1.05)	2.73 (1.38)	processing route***	*-*
Remembrance of Questionnaire	5.00 (2.07)	4.02 (2.09)	2.80 (1.54)	2.35 (1.41)	processing route* forewarning ***	
IMI	5.01 (1.11)	4.47 (0.90)	4.20 (1.11)	4.35 (0.82)	forewarning** interaction*	CR > PR in F* F > NF in CR***
Purchase Intention	2.33 (1.32)	2.78 (1.32)	3.26 (1.28)	2.67 (1.15)	forewarning* interaction*	CR > PR in NF* F < NF in CR**
Ad Attitude	2.49 (1.26)	3.22 (1.14)	3.24 (1.25)	3.11 (1.14)	forewarning^†^ interaction*	CR < PR in F** F < NF in CR**
Ad Interest	2.10 (1.32)	2.53 (1.32)	3.13 (1.28)	2.70 (1.27)	forewarning** interaction*	F < NF in CR***
Product Evaluation	2.67 (1.28)	3.12 (1.25)	3.60 (1.36)	3.25 (1.01)	forewarning** interaction*	F < NF in CR**

F = forewarned, NF = not forewarned, CR = central route, PR = peripheral route. For example, "CR > PR in F" means, in the forewarning condition, the central route score was significantly larger than that of the peripheral route condition.

^†^*p* < .10

*p < .05

**p < .01

***p < .001.

To test whether participants recognized the black background effect, we analyzed the accuracy of recognition test, which resulted in 96.20% accuracy (76 participants of 79), indicating that most participants recognized the black background effect. In addition, four graduate students who majored in psychology, including the correspondence author, coded the free-writing task on the black background effect into binary values using the following two aspects: “Did the participants recall the content of the black background effect correctly? (simple recall accuracy)” and “Did the participants regard the black background effect as a technique that they should pay attention to? (warning recall accuracy).” When the four coders’ coding was inconsistent, we adopted the majority. When the coders’ opinions were divided evenly, the conflict was resolved through discussion. As a result, the simple recall accuracy was 68.35% (54 participants of 79) and the warning recall accuracy was 64.56% (51 participants of 79). None of the recognition test accuracy, simple recall accuracy, or warning recall accuracy results were correlated with IMI: *r*(77) = .05, *p* = .646; *r* (77) = -.14., *p* = .232; *r* (77) = -.16., *p* = .163, respectively.

### Inference of manipulative intent

To test our hypothesis, we ran a 2 × 2 ANOVA on IMI. The ANOVA revealed a significant interaction effect, *F* (1,158) = 4.81, *p* = .030, *partial η*^2^ = .03, and the forewarning’s main effect, *F* (1,158) = 8.90, *p* = .003, *partial η*^2^ = .05, while no significant processing route effect emerged, *F* (1,158) = 1.61, *p* = .206 *partial η*^2^ = .01 (Table 2). A simple effect analysis also revealed that, in line with our hypothetical prediction, forewarned participants regarded the ad as more manipulative than non-forewarned participants, but only in the central route condition, *F* (1,158) = 13.03, *p* < .001, *partial η*^2^ = .08. Also, those who elaborated when reading the ad tended to rate it as more manipulative than those who did not, but only in the forewarned condition, *F* (1,158) = 5.83, *p* = .017, *partial η*^2^ = .04.

**Table 2:** Results of a hierarchical regression on IMI.

Dependent Variable = IMI		Step 1			Step 2			Step 3	
	B	*β*		B	*β*		B	*β*	
Intercept	1.88	–	*	1.98	–	*	2.08	–	**
Sex (Male = 1, Female = 0)	-0.03	-.02		0.01	.01		0.04	.02	
Age	0.00	-.03		0.00	-.03		0.00	-.03	
Need for Cognition	0.17	.16	*	0.16	.15	†	0.14	.13	†
SKEP	0.64	.41	***	0.63	.40	***	0.63	.40	***
Perceived Vulnerability to Ads	-0.11	-.11		-0.13	-.14	†	-0.14	-.14	*
Personal Involvement in Bottled Water	-0.08	-.13	†	-0.07	-.11		-0.07	-.10	
Processing Route (CR = 0.5, PR = -0.5)	–	–		0.26	.13	†	0.27	.13	†
Forewarning (F = 0.5, NF = -0.5)	–	–		0.41	.20	**	0.42	.21	**
Processing Route × Forewarning	–	–		–		–	0.62	.15	*
Adj. *R*^*2*^		.205	***		.250	***		.268	***
Δ. Adj. *R*^*2*^		–			.045	**		.018	*
*N*		162			162			162	

F = forewarned, NF = not forewarned, CR = central route, PR = peripheral route.

†*p* < .10

*p < .05

**p < .01

***p < .001.

To assess the manipulation effects in controlling other factors, we conducted a hierarchical multiple regression analysis on IMI (Table 2). We encoded the condition variables as contrast variables (0.5 or -0.5) and obtained a significant increase in adjusted *R*^*2*^ in Steps 2 and 3. In Step 3, forewarning’s main effect and the interaction effect significantly influenced IMI.

**Fig 1:**
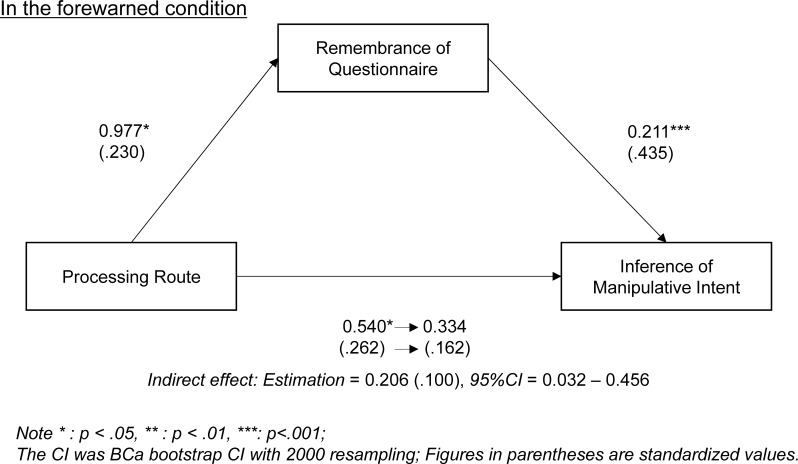
Mediation analysis: IMI mediated by remembrance of questionnaire.

Moreover, to investigate whether this difference in IMI was caused by differences in remembrance of forewarning, we conducted a mediation analysis among processing route, remembrance of questionnaire, and IMI in each forewarning condition: forewarned and control (Fig 1). First, we conducted a correlation analysis between the dummy coded processing route (central = 1, peripheral = 0) and IMI (Table 3). We observed a significantly positive correlation in the forewarned condition, *r* (77) = .26, *p* = .019, but not in the control condition, *r* (81) = -.07, *p* = .504. Then, we conducted a mediation analysis for the forewarned condition, setting remembrance of questionnaire as a mediator. When the mediation path was added, the direct coefficient from processing route to IMI was rejected, as it proved to be non-significant. Instead, the indirect effect mediated by remembrance of questionnaire became significant, meaning that the direct effect was completely mediated by remembrance of questionnaire. Therefore, we concluded that processing routes affect IMI through by remembering the forewarning, which supported our hypothesis.

**Table 3:** Zero-order correlations in each forewarning condition.

		1		2		3		4		5		6		7	
1	Processing Route (CR = 1, PR = 0)	–		.23	*	.26	*	-.17		-.30	**	-.17		-.18	
2	Remembrance of Questionnaire	.15		–		.47	***	-.30	**	-.39	***	-.37	**	-.43	***
3	IMI	-.07		-.02		–		-.58	***	-.78	***	-.60	***	-.82	***
4	Purchase Intention	.23	*	.08		-.71	***	–		.63	***	.81	***	.72	***
5	Ad Attitude	.06		.04		-.75	***	.71	***	–		.70	***	.83	***
6	Ad Interest	.16		.20	†	-.61	***	.76	***	.72	***	–		.73	***
7	Product Evaluation	.15		.07		-.77	***	.80	***	.77	***	.71	***	–	

Correlations above the diagonal are in the forewarned condition, and below the diagonal are in the control condition;

CR = central route, PR = peripheral route;

†p < .10

*p < .05

**p < .01

***p < .001.

### Additional analyses

To investigate whether the conditions affect remembering the questionnaire, we ran a 2 × 2 ANOVA on remembrance of questionnaire (Table 1). The result indicated that the main effect of processing route and forewarning was significant, *F* (1,158) = 6.35, *p* = .013, *partial η*^2^ = .04; *F* (1,158) = 46.64, *p* < .001, *partial η*^2^ = .23; respectively, but the interaction effect was insignificant, *F* (1,158) = 0.85, *p* = .356, *partial η*^2^ = .01. This result reveals that central route processing encourages remembering the questionnaire even in the control condition.

Moreover, we performed a 2 × 2 ANOVA on each ad evaluation scale and observed the same pattern: the interaction effect was significant in each scale (Table 1). The simple effects of forewarning in the central route were seen in all measurements, suggesting that the effect will be robust across measurements.

**Fig 2:**
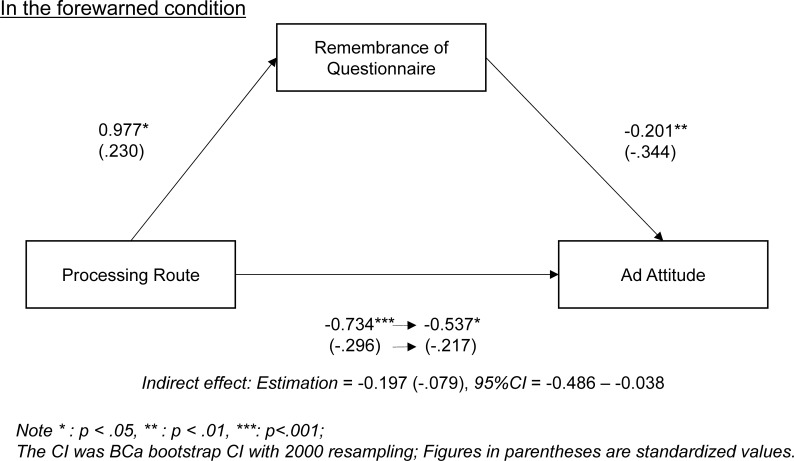
Mediation analysis: Ad attitude mediated by remembrance of questionnaire.

Identical to the mediation analysis on IMI, we conducted a mediation analysis on ad evaluations. First, we attempted a correlation analysis between dummy coded processing route and ad evaluations (Table 3). In the forewarned condition, only ad attitude had a significant correlation, *r* (77) = -.30, *p* = .008, while the other ad evaluations did not, *r*s (77) = -.18 –-.17, *p*s > .120. In the control condition, only purchase intention had a significant correlation, *r* (77) = .23, *p* = .033, while the other ad evaluations did not, *r*s (77) = .06 –.16, *p*s > .145. Then, we conducted a mediation analysis on ad attitude in the forewarned condition. The results demonstrated that ad evaluation was partially mediated by remembrance of questionnaire in the forewarned condition (Fig 2). This result suggests that participants partially lessened ad evaluation by remembering the forewarning.

**Fig 3:**
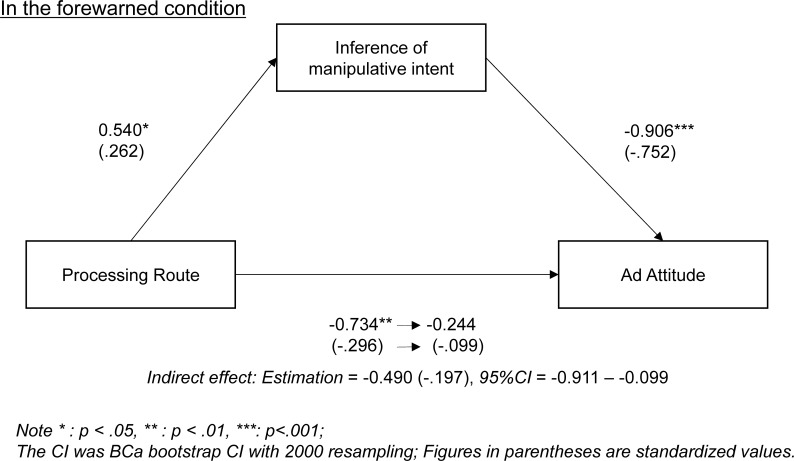
Mediation analysis: Ad attitude mediated by IMI.

To investigate the relationship between IMI and ad attitude, we attempted another mediation analysis that set IMI as a mediator between dummy coded processing route and ad attitude. The result showed that IMI completely mediated the relationship between dummy coded processing route and ad attitude (Fig 3). The relationship between dummy coded processing route and IMI was completely mediated by remembrance of forewarning, suggesting that remembering forewarnings stimulates IMI directly and as a result, ad attitude lessens indirectly.

## Discussion

In this study, we predicted that forewarned people fell victim to scams, because they did not remember the forewarning due to con artists’ appeal to emotion techniques. By replicating a real situation in the experiment, we tested the hypothesis that peripheral route processing prevents counterarguing by hindering remembrance of the forewarning. The results supported our hypothesis and suggested that the processing route affects the effect of forewarnings when deception attempts occur.

First, the interaction effect of the experimental conditions had an influence on IMI. Only forewarned and centrally processing participants rated the advertisement as more manipulative, while peripherally processing participants did not, despite being forewarned. This result suggests that a forewarning’s effectiveness depends on central route processing. We did not use an experimental situation where people could generate counterarguments in advance, rather they had to produce these counterarguments during the message presentation. Even in such situations, forewarned and centrally processing participants seemed to resist the made-up advertisement, as is consistent with prior research, which enabled them to generate anticipatory counterarguments [15]. In accordance with Petty and Cacioppo [11], this implies that counterargumentation theory can be applied not only to anticipatory counterarguments, but also to those that may occur during a message presentation.

Second, the processing route’s effect in the forewarned condition was mediated by remembrance of the questionnaire, in this case, the forewarning. According to this result, the difference in IMI rating—participants with central processing rated it higher than those with peripheral processing—suggests that the central route accelerated forewarning remembrance while the peripheral route discouraged it. Additionally, in the ANOVA results, we found the processing route’s main effect: using the central route accelerated the recollection of relevant information on the advertisement regardless of the forewarning condition. This suggests that to process information through the central route encourages relevant information recollection and that people remember forewarnings.

Although we found clear patterns on IMI, the patterns in ad evaluations were a little more ambiguous. For example, in the forewarned condition, a significant correlation with processing route only emerged in ad attitude. These unclear patterns might be a result of the advertisement itself. In the preliminary study, we selected the advertisement based on the IMI score to avoid the compounded effect with the processing route, but we did not consider the ad evaluation scores. In other words, the advertisements themselves might have an influence on ad evaluations in a specific processing route condition. For example, we found a significantly positive correlation between purchase intention and processing route in the control condition, suggesting that the advertisement only stimulated purchase intention in the central route condition, because of its potentially strong argument. These slightly different patterns from those seen in IMI were thought to derive from advertisement selection procedures. These are not major problems when we consider that significant interaction effects were consistent across all ad evaluation measures.

Considering the interpretations above, a potential reason why forewarned people are susceptible to scams is that they are not in situations in which they can remember forewarnings, which leads to counterarguing. As Kircanski et al. [13] suggested, con artists often use emotional arousal techniques that might prevent people from contemplating and remembering forewarnings. Therefore, to solve scam problems, it may be important to consider how people can process information deeply when being deceived in addition to preemptively forewarning potential victims.

It is difficult to propose a concrete strategy based on the current information, but we can refer to Boush, Friestad, and Wright [22] who placed an importance on consumers’ self-protection, and proposed that they acquire the skills to recognize and confront deceptive marketing. Similarly, perhaps potential scam victims need to learn how to protect themselves not only by receiving forewarnings, but also by practicing resisting deception attempts. Sagarin et al. [8] demonstrated that educating consumers on how to protect themselves enhances resistance to deceptive persuasion attempts. Such practice might spare the cognitive resources necessary for central processing. In any case, we need to establish new official solutions besides the traditional forewarning strategy.

### Limitations and future research

The results of our research are still inconclusive and should be interpreted with caution. First, the interaction’s effect size was very small, as it explained only 2% of the variance in the regression model and a small partial eta squared in the ANOVA model. This suggests that the processing route would not be a primary factor in determining a forewarning’s effect. Furthermore, we did not obtain a significant effect in the pilot study posted in the online supplement (S1 File), although it was extremely under-powered in its sample size. We may have disregarded other conclusive factors considering the main study had a small effect size despite its sample size.

Second, the remembrance of questionnaire, an important variable in this research, may be biased, as this was a self-reported measure. In our experimental design, participants recognized that the questionnaire, which they were told was not relevant to the experiment, was actually relevant when they saw the advertisement using the black background effect. Therefore, we cannot deny the possibility that participants answered this question based on the demand characteristic when they were asked how they remembered the questionnaire. In light of this, the mediating effect of our result should be evaluated carefully.

Third, we did not directly measure counterargumentation. Previous studies have used thought listing to measure this aspect [11, 15], but we only measured the inference of manipulative intent and ad evaluations. We can reduce the amount of generated counterarguments from the scores of such items, but ideally, this should be verified through direct measurements.

Finally, it is still questionable whether our experimental method precisely describes real scam victimization. For example, we used inference of manipulative intent for a bogus advertisement as the dependent variable, but it did not cover all types of scams. Depending on the scam type [23], different psychological processes can be assumed. Thus, we should further consider which kinds of scams can be applicable to our results.

In summary, our results are not yet compelling, although the interaction between processing route and forewarning is consistent with other work [11] [15]. Therefore, in future research, we need to verify the robustness of our findings by changing the manipulation or variable types.

## Supporting information

S1 File Preliminary survey and pilot study. (DOCX)

S2 File Preliminary survey data. (CSV)

S3 File Pilot study data. (CSV)

S4 File Main study data. (CSV)
