# Supplementary material for: Effective forewarning requires central route processing: Theoretical improvements on the counterargumentation hypothesis and practical implications for scam prevention
Source: PLoS One. 2020 Mar 5;15(3):e0229833. doi: 10.1371/journal.pone.0229833 (PMC7058298; doi:10.1371/journal.pone.0229833)
Supplement: S1 File — (DOCX) [file pone.0229833.s001.docx]

# Preliminary survey

### Participants

Fifty-eight college students (34 males and 24 females, *M_age_* = 20.38±1.02, *Range*: 19–23) in Osaka, Japan evaluated imaginary advertisements composed of sentences without pictures. Before the psychology class began, we asked the students to join our free survey, and only students who agreed participated. Based on randomly assigned ads, half the participants read two of the four advertisements (Ads A and B), and the other half read the other two (Ads C and D). We split the four advertisements to prevent participants from answering too many questions. As a result, 29 participants evaluated Ads A and B (13 males and 16 females, *M_age_* = 20.48±1.18, *Range*: 19–23) and the other 29 participants evaluated Ads C and D (11 males and 18 females, *M_age_* = 20.28±0.84, *Range*: 19–22). They evaluated the ads on a seven-point Likert scale. The ads were about bottled water (Ads A and D), a toothbrush (Ad B), and juice (Ad C), with 489 characters (Ad A), 173 characters (Ad B), 279 characters (Ad C), and 411 characters (Ad D) in Japanese. The survey took approximately 15 minutes to complete.

## Measurements

We measured ad evaluations in an identical way to the main study: purchase intention, ad attitude, product evaluation, ad interest, and inference of manipulative intent (IMI) [18]. In addition, participants indicated responses regarding their levels of elaboration for each ad through the following item: “How well did you concentrate when you read the first (or second) advertisement?” (answered on a seven-point scale ranging from 1 = “not at all” to 7 = “extremely”). In the following analysis, this measure was used for dividing the participants into two groups of the central route and the processing route.

## Results

There were no advertisements that exhibited a prominent ceiling or floor effect in IMI (Table 1). As we were unable to manipulate the processing route in advance, we separated the participants into two groups depending on their scores for the level of elaboration for each advertisement. We regarded participants whose scores were below the mean as peripheral route group members, and those above the mean as central route group members. We then examined whether the IMI score differed significantly between the central and peripheral route groups for each advertisement. For Ad A, *t* (22.84) = 0.99, *p* = .334, *d* = 0.37, and Ad B, *t* (10.15) = 1.29, *p* = .227, *d* = 0.60, there was no significant difference between the two groups, whereas Ad C, *t* (24.95) = 1.92, *p* = .067, *d* = 0.72, and Ad D, *t* (24.76) = 2.33, *p* = .028, *d* = 0.85, showed a significant difference (Table 2). Our aim in this preliminary survey was to choose a message that was not affected by the processing route; therefore, we chose Ad A as the stimulus because it had the smallest effect size that was insignificant.

**Table 1. Means (SDs) of ad evaluations**

|  | Ad A | | | | Ad B | | | |
| --- | --- | --- | --- | --- | --- | --- | --- | --- |
|  | *Mean* | *SD* | *Median* | *α* | *Mean* | *SD* | *Median* | *α* |
| IMI | 3.85 | 0.98 | 4.00 | .82 | 4.49 | 1.03 | 4.33 | .84 |
| Purchase Intention | 4.21 | 1.10 | 4.33 | .63 | 3.79 | 1.25 | 4.00 | .87 |
| Ad Attitude | 3.62 | 1.15 | 3.67 | .71 | 3.75 | 0.94 | 4.00 | .64 |
| Ad Interest | 3.49 | 1.22 | 3.33 | .61 | 3.95 | 1.09 | 4.00 | .55 |
| Product Evaluation | 4.29 | 1.27 | 4.50 | .89 | 3.78 | 1.19 | 3.75 | .88 |
|  |  |  |  |  |  |  |  |  |
|  | Ad C | | | | Ad D | | | |
|  | *Mean* | *SD* | *Median* | *α* | *Mean* | *SD* | *Median* | *α* |
| IMI | 3.80 | 1.02 | 3.83 | .78 | 3.64 | 1.05 | 3.67 | .87 |
| Purchase Intention | 4.12 | 1.60 | 4.00 | .86 | 4.07 | 1.41 | 4.00 | .77 |
| Ad Attitude | 3.86 | 1.11 | 4.00 | .64 | 4.08 | 1.11 | 4.00 | .72 |
| Ad Interest | 3.40 | 1.20 | 3.33 | .64 | 3.95 | 1.57 | 4.33 | .86 |
| Product Evaluation | 3.82 | 1.20 | 3.75 | .74 | 4.24 | 1.22 | 4.25 | .85 |

**Table 2. Means (SDs) of manipulation checks divided by level of elaboration**

|  | Central Route | | |  | Peripheral Route | | | *d* |  |
| --- | --- | --- | --- | --- | --- | --- | --- | --- | --- |
|  | *N* | *Mean* | *SD* |  | *N* | *Mean* | *SD* |  |  |
| Ad A | 18 | 3.71 | 1.02 |  | 11 | 4.08 | 0.93 | 0.37 |  |
| Ad B | 21 | 4.32 | 0.92 |  | 8 | 4.94 | 1.23 | 0.61 |  |
| Ad C | 15 | 3.47 | 0.87 |  | 14 | 4.17 | 1.08 | 0.72 | † |
| Ad D | 15 | 3.23 | 1.14 |  | 14 | 4.07 | 0.78 | 0.85 | ** |
| *Note:* † *p* < .10, * *p* < .05, ** *p* < .01, *** *p* < .001 | | | | | | | | | |

# Pilot experiment

## Method

Participants

Seventy-three undergraduate or graduate university students in Japan (34 male and 39 female participants, *M_age_* = 21.66±2.81, *Range*: 19–37) participated for a bookstore gift card worth JPY 500.

Design

A 2 (forewarning: forewarned or control) × 2 (processing route: central or peripheral) between-participants design was adopted.

Procedure

The procedure was almost the same as the main study, but they differed at some points because this experiment was conducted in a laboratory. After entering the experiment room, participants received a fake explanation about the experiment. First, the experimenter informed the participant that this experiment was aimed at investigating the effect of task performance on buying behavior, but that another researcher had asked him to administer a simple questionnaire. Following this explanation, participants were asked to answer a questionnaire that they were told was not related to this experiment. The questionnaire was almost the same as that which was used in the main study. It outlined six general psychological phenomena: “misattribution of arousal,” “ingroup favoritism,” “black background effect (or veil effect),” “bystander effect,” “correspondence bias,” and “Zeigarnik effect.” Participants then rated the phenomena using the following three items rated on a seven-point scale: “I know this phenomenon,” “I am interested in this phenomenon,” and “I would like to learn more about this in psychology class.” The forewarning condition was manipulated at that time. In the forewarning condition, a fake psychology phenomenon of the “black background effect” was included in the six psychological phenomena. The explanation of the black background effect was the same as that in the main study: “People generally become gullible when they encounter white letters used on a black background because this combination makes people less perceptive. Therefore, people should be wary of this as some con artists use this technique.” Instead, participants in the control condition received an explanation of the “veil effect” which was also an imaginary psychological term created for this experiment that was unrelated to the experiment. The veil effect was explained as follows: “The veil effect depicts the tendency of human beings to want to know hidden things. For example, people want to enter a place where a signboard says to ‘keep out.’” The black background effect was related to the stimuli that they were going to see later.

After participants filled out the questionnaire, the experimenter pretended to begin his own experiment and instructed participants to perform a calculation task that included 20 basic arithmetic questions. The calculation task’s aim was to distract participants from the previous questionnaire. Thirty seconds after finishing the calculation task, an imaginary advertisement showed up on the screen, and participants were asked to read it. The advertisement was about bottled water, and was composed of white letters on a black background (the black background effect). The ad was identical to Ad A in the preliminary survey. The advertisement was presented for either 40 seconds in the peripheral route condition, or 120 seconds in the central route condition. In addition, in the central route condition, participants were asked to concentrate on the ad. These stipulations were intended to manipulate motivation (instruction) and the ability (time limit) to elaborate following the assumption of ELM by Petty and Cacioppo [12].

Upon completion of reading the ad, participants answered a questionnaire about it. At the end of the experiment, the experimenter debriefed the true aim of the experiment and apologized to the participants for using deception.

### Measurements

Participants rated the items for “advertisement evaluations,” “manipulation checks,” and “personalities” in this order.

Advertisement evaluations

We measured purchase intention, ad attitude, product evaluation, ad interest, and IMI. They were completely identical to the items used in the main study.

Manipulation checks

To determine whether the manipulation changed the information processing route, we asked participants to indicate their level of elaboration and remembrance of the questionnaire, which were identical to the main study. Additionally, we measured the perceived difficulty of the calculation task using three items rated on a seven-point scale: “The calculation task was difficult,” “I did not feel taxed by the task (reversal),” and “The task racked my brain.” Then, using a dichotomous scale, we asked them whether they were aware of the bottled water before the experiment started. Subsequently, we conducted recognition and recall tests. The measurements were almost the same as those in the main study, but the target term in the control condition was different—the veil effect was an imaginary term—but the content seemed plausible.

Personalities

Participants answered the Japanese version of the need for cognition scale [19] and skepticism toward advertising scale (SKEP) [20], which were also identical to the main study.

## Results

Participants who ignored the instructions (one participant), who were not native Japanese speakers (two participants), or who answered that they had known about the black background effect (four participants, including one participant who was not a native Japanese speaker) were excluded from the analysis. No one answered that they knew the imaginary bottled water in the advertisement. Finally, we analyzed 67 participants’ data (32 males and 35 females, *M_age_* = 21.46±2.58, *Range*: 19–37).

Manipulation check

To test whether the manipulation in the experiment changed the information processing route, we ran a 2 × 2 analysis of variance (ANOVA) on the participants’ reported levels of elaboration (*α* = .85). As we expected, only the main effect of the processing route change emerged as significant, *F* (1, 63) = 15.30, *p* < .001, *partial* *η*^2^ = .20. The central-route processing respondents reported a higher level of elaboration than the peripheral-route processing respondents (Table 3), but the scores were higher than the middle point (four), even in the peripheral route condition.

**Table 3. Means (SDs) of manipulation checks and ad evaluations**

|  | Forewarned | |  | Not Forewarned | |  |
| --- | --- | --- | --- | --- | --- | --- |
|  | Central | Peripheral |  | Central | Peripheral |  |
| Level of Elaboration | 5.73 (0.76) | 4.56 (1.38) |  | 5.39 (0.80) | 4.46 (1.31) |  |
| Remembrance of Questionnaire | 3.88 (2.27) | 4.03 (2.51) |  | 1.62 (0.88) | 1.42 (0.49) |  |
| IMI | 4.42 (0.75) | 4.40 (0.76) |  | 4.30 (0.95) | 4.13 (0.99) |  |
| Purchase Intention | 3.86 (1.37) | 3.13 (1.08) |  | 3.84 (1.44) | 3.07 (1.13) |  |
| Ad Attitude | 3.69 (0.96) | 3.51 (0.92) |  | 3.86 (1.12) | 3.78 (0.98) |  |
| Ad Interest | 3.71 (1.17) | 3.16 (1.01) |  | 3.41 (1.28) | 3.24 (1.02) |  |
| Product Evaluation | 4.31 (1.06) | 4.12 (1.10) |  | 4.15 (1.05) | 4.11 (1.11) |  |

Thereafter, to test whether participants recognized the black background effect, we examined the accuracy of the recognition test. The recognition test on the black background effect had 100% accuracy (32 of 32 participants), indicating that all participants recognized the term. In addition, we calculated the simple recall accuracy and warning recall accuracy in the same way as in the main study. As a result, the simple recall accuracy was 96.88% (31 of 32 participants) and the warning recall accuracy was 84.38% (27 of 32 participants). However, neither the simple recall accuracy nor the warning recall accuracy correlated with IMI, where *r* (30) = -.27, *p* = .14; and *r* (30) = -.07, *p* = .70, respectively. In summary, it was proved that the participants recognized and remembered the black background effect on demand and that they regarded it as a technique to remember, but the indices did not affect the inference of manipulative intent.

Advertisement evaluations

To test the hypotheses, we ran a 2 × 2 ANOVA on IMI (*α* = .85). The ANOVA revealed no main effects or interaction effects, *F*s < 0.82, *p*s *>* .367, *partial* *η*^2^s < .01 (Table 1). Moreover, the 2 × 2 ANOVA on the other advertisement evaluations (*α*s = .74 - .85) revealed the only significant main effect as the processing route on purchase intention, *F* (1, 66) = 5.84, *p* = .018, *partial* *η*^2^ = .08, whereas no other effects were seen.

To assess the true manipulation effects in controlling other factors, we conducted a multiple regression analysis toward IMI (Table 4). The condition variables were encoded as contrast variables (0.5 or -0.5) to avoid multicollinearity. Moreover, the alpha of the calculation task difficulty was quite low (*α* = .40); we used only the item “The calculation task was difficult for me” for this regression. The result revealed the only significantly positive effect as SKEP on IMI, while the condition variables did not have an effect.

**Table 4. Results of multiple regression on IMI**

| Dependent Variable = IMI | B | *b* | |
| --- | --- | --- | --- |
| Intercept | 1.88 | – |  |
| Sex (Male = 1, Female = 0) | -0.08 | -.04 |  |
| Age | 0.01 | .03 |  |
| Calculation Task Difficulty | 0.12 | .20 |  |
| Need for Cognition | 0.03 | .03 |  |
| SKEP | 0.47 | .29 | * |
| Processing Route (CR = 0.5, PR = -0.5) | -0.02 | -.01 |  |
| Forewarning (F = 0.5, NF = -0.5) | 0.30 | .17 |  |
| Processing Route × Forewarning | -0.29 | -.09 |  |
| Adj. *R^2^* | .047 | *n.s.* | |
| *Note:* F = forewarned, NF = not forewarned, CR = central route, PR = peripheral route. †*p* < .10, **p* < .05, ***p* < .01, ****p* < .001., *n.s.* = non-significant. | | | |

Remembrance of questionnaire

To investigate whether the conditions affected the remembrance of the questionnaire, we ran a 2 × 2 ANOVA (Table 3). The result indicated that only the main effect of forewarning was significant, *F* (1, 63) = 33.40, *p* < .001, *partial* *η*^2^ = .35; whereas the other effects were insignificant, *F*s < 0.17, *p*s *>* .678, *partial* *η*^2^s < .01.

## Discussion

Through the pilot study, we found three major problems which may have prevented the interaction effect of IMI. First, the manipulations of the processing route seemed to be inappropriate. Even in the peripheral route condition, the level of elaboration score was higher than the middle point: four. Therefore, the manipulation may not have deprived the participants in the peripheral route of their motivation and ability to think, potentially causing no significant manipulation effects on IMI. Thus, we changed the way of manipulating the processing route in the main study.

Second, it was possible that the calculation task disturbed the recall of forewarning more than expected. We asked participants to perform this task to distract them from noticing the true aim of the experiment, but this task may have completely cut the link between the black background effect and the advertisement shown later. In fact, we did not find the significant effect of the processing route or the interaction effect on remembrance of forewarning. This result means that the participants could not remember the forewarning even when they centrally processed the advertisement. In other words, participants were unable to remember the black background effect spontaneously due to the considerably stressful calculation task, although they could remember it on demand, considering the substantially high accuracy rates in the recognition and recall tests. Therefore, in the main study, we deleted the calculation task.

Finally, our sample size was quite small, and the participants’ attribution was biased toward university students. This caused underpowered statistical tests and biased results. Therefore, in the main study, we collected the data online to acquire a sufficient sample size and to not limit our participants to only university students.
